# Temporal Changes in Biochemical Responses to Salt Stress in Three *Salicornia* Species

**DOI:** 10.3390/plants13070979

**Published:** 2024-03-29

**Authors:** Hengameh Homayouni, Hooman Razi, Mahmoud Izadi, Abbas Alemzadeh, Seyed Abdolreza Kazemeini, Ali Niazi, Oscar Vicente

**Affiliations:** 1 Department of Plant Production and Genetics, School of Agriculture, Shiraz University, Shiraz 71946-84471, Iran; hengamehomayouni@gmail.com (H.H.); izadi2005@gmail.com (M.I.); alemzadeh@shirazu.ac.ir (A.A.); akazemeini@shirazu.ac.ir (S.A.K.); 2 Institute of Biotechnology, Shiraz University, Shiraz 71468-64685, Iran; niazi@shirazu.ac.ir; 3 Institute for the Conservation and Improvement of Valencian Agrodiversity (COMAV), Universitat Politècnica de València, Camino de Vera s/n, 46022 Valencia, Spain

**Keywords:** *Salicornia persica*, *Salicornia europaea*, *Salicornia bigelovii*, glycine betaine, ascorbate peroxidase, halophytes

## Abstract

Halophytes adapt to salinity using different biochemical response mechanisms. Temporal measurements of biochemical parameters over a period of exposure to salinity may clarify the patterns and kinetics of stress responses in halophytes. This study aimed to evaluate short-term temporal changes in shoot biomass and several biochemical variables, including the contents of photosynthetic pigments, ions (Na^+^, K^+^, Ca^2+^, and Mg^2+^), osmolytes (proline and glycine betaine), oxidative stress markers (H_2_O_2_ and malondialdehyde), and antioxidant enzymes (superoxide dismutase, peroxidase, catalase, and ascorbate peroxidase) activities of three halophytic *Salicornia* species (*S. persica*, *S. europaea*, and *S. bigelovii*) in response to non-saline, moderate (300 mM NaCl), and high (500 mM NaCl) salinity treatments at three sampling times. *Salicornia* plants showed maximum shoot biomass under moderate salinity conditions. The results indicated that high Na^+^ accumulation in the shoots, coupled with the relative retention of K^+^ and Ca^2+^ under salt stress conditions, contributed significantly to ionic and osmotic balance and salinity tolerance in the tested *Salicornia* species. Glycine betaine accumulation, both constitutive and salt-induced, also seems to play a crucial role in osmotic adjustment in *Salicornia* plants subjected to salinity treatments. *Salicornia* species possess an efficient antioxidant enzyme system that largely relies on the ascorbate peroxidase and peroxidase activities to partly counteract salt-induced oxidative stress. The results also revealed that *S. persica* exhibited higher salinity tolerance than *S. europaea* and *S. bigelovii*, as shown by better plant growth under moderate and high salinity. This higher tolerance was associated with higher peroxidase activities and increased glycine betaine and proline accumulation in *S. persica.* Taking all the data together, this study allowed the identification of the biochemical mechanisms contributing significantly to salinity tolerance of *Salicornia* through the maintenance of ion and osmotic homeostasis and protection against oxidative stress.

## 1. Introduction

While the world’s population is constantly increasing, adverse environmental factors contribute to reducing arable land, making future food security a challenging goal to achieve. High soil salinity has become a growing global problem that seriously threatens sustainable food and feed production. Approximately 10^9^ hectares of the world’s land area (about 7% of total world land) are currently affected by salinity [1]. High salinity has deleterious effects on the physiological and biochemical processes of plants, leading to limited growth and productivity and, eventually, plant death.

One effective strategy to deal with soil salinity is to breed and cultivate halophyte plant species with economic value in saline marginal lands or abandoned cropland affected by secondary salinisation. Halophytes are plants of natural saline habitats [2] that can tolerate high salinity and may even need such environments for optimal growth [3]. Halophytes can be used for food, fodder, biofuels, and soil phytoremediation [4]. Some are also used as medicinal plants or for pharmaceutical purposes because they contain biologically active compounds such as terpenes, phenols, antioxidants, and biological antibacterials [5]. Moreover, an in-depth understanding of the salinity tolerance mechanisms of halophytes can provide valuable scientific insights into the biotechnological improvement of crop resilience against salinity stress. Halophytes employ both short-term responses and long-term adaptive mechanisms to survive and grow in saline environments. Short-term responses often involve rapid biochemical adjustments, such as accumulation of compatible solutes, regulation of ion transport, and activation of antioxidant systems to mitigate immediate osmotic and oxidative stresses. On the other hand, long-term adaptive mechanisms include morphological and physiological changes such as enhanced root architecture or, in some species, the formation of salt glands to sustain plant growth and reproduction under high salinity conditions [6]. Indeed, it is crucial to elucidate how the rapid biochemical responses dynamically evolve as the duration and intensity of salt stress change, allowing halophytes to eventually acquire long-term adaptations to thrive in saline environments.

*Salicornia*, known by the common names of glasswort or pickleweed, is a genus of halophyte plants belonging to the Amaranthaceae family [7]. These herbaceous plants have medicinal, industrial, and edible properties; they commonly grow in saline soils of coastal areas and the margins of inland salt marshes or salt lakes [8]. *Salicornia* species are widely distributed in North America, Europe, and Central and South Asia [7]. Members of this genus do not possess salt bladders or salt glands, and thus they are considered models for dissecting the common mechanisms underlying plant tolerance to salinity [9]. Different tolerance mechanisms such as the regulation of transport and compartmentalisation of toxic ions, energy homeostasis, osmotic adjustment by cytosolic accumulation of osmolytes, and detoxification of excessive levels of reactive oxygen species (ROS) by activating antioxidant systems are utilised by the *Salicornia* species to adapt to high salt concentrations [10,11,12,13].

Halophytes are equipped with effective enzymatic and non-enzymatic antioxidant systems to cope with high salinity [14]. Antioxidant enzymes such as peroxidase (POD), catalase (CAT), superoxide dismutase (SOD), and ascorbate peroxidase (APX) play key roles in counteracting the generation of ROS induced by high salinity. Plants with higher antioxidant levels, whether constitutive or induced, have shown greater tolerance to oxidative damage [15]. There are few reports on the enzymatic antioxidant system of *Salicornia* species activated in response to salt treatments. Salinity significantly enhanced the activities of SOD, CAT, and POD in *Salicornia persica* and *Salicornia europaea* [16,17]. Similarly, salinity treatments caused an increase in the activities of APX, SOD, and glutathione reductase (GR) in *Salicornia brachiata* shoots [18]. On the other hand, a decline in CAT activity was observed in *S. brachiata* and *S. europaea* under salinity conditions [18,19]. Oxidative stress induced by salinity can also affect the photosynthetic rate by altering photosynthetic pigment contents. Some halophytic species are capable of maintaining chlorophylls and carotenoids in saline environments [20,21]; however, *Salicornia* species have shown reduced levels of photosynthetic pigments in response to salt treatments [19,22].

Accumulation of osmolytes such as proline, glycine betaine, polyphenols, and soluble sugars is a common defence mechanism of halophytes to tackle high-salinity conditions [23]. Increased proline content is a typical adaptive response observed in many plant species as soon as stress occurs [24]. Glycine betaine is another essential osmoprotectant that can be accumulated in response to salinity [25]. Glycine betaine can maintain cellular turgor pressure to prevent oxidative stress [26]. Previous reports demonstrated that high salinity induced a significant increase in proline [16,18,20] and glycine betaine [27] contents in *S. europaea*. Also, in *S. persica*, proline and glycine betaine concentrations increased with increasing salt concentrations [28].

Halophytes utilise ion homeostasis as a central mechanism to maintain cellular water balance through osmotic adjustment under saline conditions [29]. *Salicornia* species do not act as salt excluders; on the contrary, they dramatically accumulate Na^+^ in shoots in response to salt treatments [21]. A high uptake of Na^+^ can alter the composition of other inorganic ions, particularly potassium, calcium, and magnesium, which are major components of signalling pathways involved in salinity tolerance. Increased salt concentrations decreased the amounts of K^+^, Ca^2+^, and Mg^2+^ in shoots of *Salicornia herbacea* [30,31] and *S. persica* [28]. A reduction in K^+^ content was also observed under varying NaCl concentrations in other *Salicornia* species, including *S. brachiata* [18] and *S. europaea* [16,27]. However, another study in *S. europaea* reported an increase in shoot K^+^ concentration under moderate salinity, whereas Na^+^ and Ca^2+^ contents increased significantly at all tested NaCl concentrations compared to control plants grown without salt [20].

Less is known about the differences in salinity tolerance in *Salicornia* species. Few studies have compared the biochemical reactions of different *Salicornia* species under salinity [17,21]. For instance, *S. persica*, which is native to central and south-central Iran [32], showed a higher level of tolerance compared to *S. europaea*, possibly because of more efficient protection against oxidative damage through ion homeostasis and antioxidant activities [17]. It is presumed that salinity-induced biochemical responses get altered with passing time, so multiple measurements of biochemical attributes during exposure to salinity may elucidate the patterns and kinetics of plant stress responses. Time-course evaluations of changes in biochemical variables may clarify some aspects of how *Salicornia* species can adapt so efficiently to high salinity over time, gaining insights into the biochemical pathways and regulatory mechanisms involved in tolerance to salt stress. No reports, other than that of Parida and Jha [18] on *S. brachiata*, have yet focused on the temporal changes of biochemical responses of *Salicornia* species under varying salinity conditions.

The present time-course study aimed to illuminate the short-term temporal variation of biochemical parameters related to salt stress responses in plants of three *Salicornia* species, *S. europaea*, *S. bigelovii*, and *S. persica*, subjected to NaCl treatments. Therefore, shoot biomass and water content and a collection of biochemical stress biomarkers, including photosynthetic pigments (chlorophylls and carotenoids), ions (Na^+^, K^+^, Ca^2+^, and Mg^2+^), osmolytes (proline and glycine betaine), oxidative stress markers (malondialdehyde (MDA) and H_2_O_2_), and antioxidant enzymes (SOD, POD, CAT, and APX) activities were determined at different times (one, three, and eight days) after starting the treatments with three different salt concentrations, (0, 300, and 500 mM NaCl). The obtained data were subjected to various statistical analyses to better understand the contribution and significance of the biochemical responses in salinity tolerance mechanisms of the tested *Salicornia* species.

## 2. Results

### 2.1. Growth Parameters

The effects of salinity on the growth of the three selected *Salicornia* species, assessed using fresh biomass and water content of the shoots, were investigated at three sampling times. The analysis of variance showed that the different *Salicornia* species, salinity treatments, and sampling times had highly significant effects on the fresh weight and water content of the plant shoots. The interactions “Species × Salinity”, “Species × Time”, and “Salinity × Time” were also significant for both parameters, whereas the triple interaction “Species × Salinity × Time” was significant for fresh weight but not for water content (Table 1).

**Figure 1:**
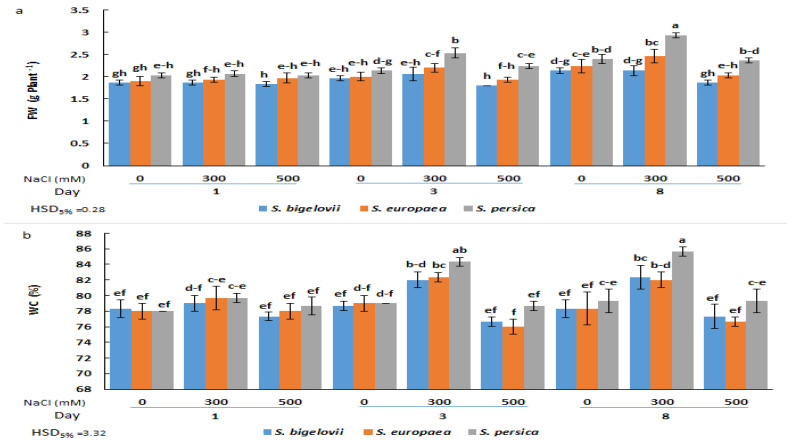
Effects of salt treatments (0, 300, and 500 mM NaCl) on the fresh weight (FW) (**a**) and water content (WC) (**b**) of the three selected *Salicornia* species (*S. bigelovii*, *S. europaea*, and *S. persica*) at three sampling times (1, 3, and 8 days after starting the treatments). The values are means ± SD. Different lowercase letters over the bars indicate significant differences between mean values; HSD values (*p* ≤ 0.05) are included for comparing the means.

No significant differences in FW were observed one day after starting the NaCl treatments, neither between species nor between salt concentrations, although mean FW values were slightly higher for *S. persica* than for *S. europea* and *S. bigelovii* at all salinity levels (Figure 1a). However, after three or eight days of treatment, the FW of *S. persica* plants grown under moderate salinity (300 mM NaCl) was significantly higher than that of *S. europea* and *S. bigelovii* plants and also higher than in the control, non-treated plants. Under severe salinity conditions (500 mM NaCl), both in the three-day and eight-day samples, shoot biomass was reduced to control values in the three species, although the significant difference between *S. persica* and the other two *Salicornia* species was maintained (Figure 1a). Changes in the shoot water content percentages showed a similar qualitative pattern, with significant increases over control values (plants grown in the absence of salt) only in the 300 mM NaCl treatments. However, in this case, a significantly higher WC in *S. persica* plants compared to the other two species was only detected after eight days of treatment (Figure 1b). These data suggest that the observed increase in shoot fresh biomass at moderate salinity was due, at least in part, to an increase in plant succulence.

### 2.2. Photosynthetic Pigments Contents

**Figure 2:**
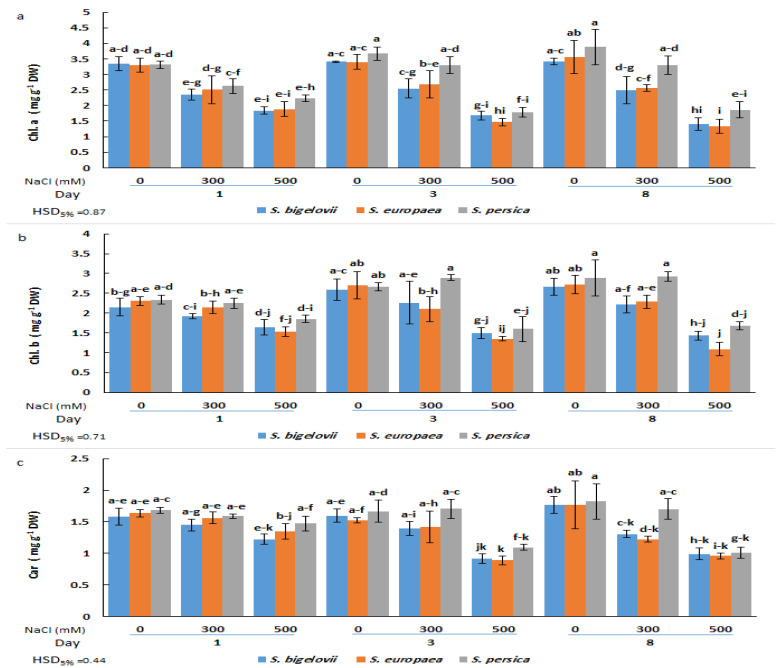
Effects of salt treatments (0, 300, and 500 mM NaCl) on chlorophyll a (Chl. A) (**a**), chlorophyll b (Chl. B) (**b**), and carotenoids (Car) (**c**) of the three selected *Salicornia* species (*S. bigelovii*, *S. europaea*, and *S. persica*) at three sampling times (1, 3, and 8 days after starting the treatments). The values are means ± SD. Different lowercase letters over the bars indicate significant differences between mean values; HSD values (*p* ≤ 0.05) are included for comparing the means.

The analysis of variance illustrated the significant effects of the salinity levels and the *Salicornia* species on the contents of chlorophyll a (Table 1). A significant and rapid decrease in chlorophyll a was observed in plants of the three *Salicornia* species subjected for one day to high salinity treatment; no further significant reductions in the contents of chlorophyll a were detected at subsequent sampling times after three and eight days of treatment. Minimum chlorophyll a contents (about 60% reduction compared to the corresponding controls) were measured in *S. europaea* and *S. bigelovii* plants after eight days of applying 500 mM NaCl, although the observed temporal changes in chlorophyll a contents were not statistically significant. On the other hand, the ANOVA results also showed significant salinity by time interaction, implying that the effect of salinity levels on chlorophyll a contents varied with the sampling times. The *Salicornia* species showed a different response pattern for the contents of chlorophyll a under moderate salinity; *S. persica* chlorophyll a levels were similar to those measured under non-saline conditions, whereas the concentrations in the other *Salicornia* species significantly declined after eight days of treatment with 300 mM NaCl (Figure 2a).

Chlorophyll b and carotenoid contents were significantly affected by the salinity treatments, the *Salicornia* species, and the sampling times (Table 1). Chlorophyll b concentrations did not exhibit statistically significant differences among plants of the three *Salicornia* species under any tested experimental condition. In the presence of 300 mM NaCl, the plants could maintain the chlorophyll b contents at similar levels as non-stressed plants at all sampling times. However, these levels decreased significantly for the three species three days after high salinity exposure and remained unchanged at the last sampling time. The minimum chlorophyll b content (53% reduction compared to the non-stressed control) was measured in *S. europaea* after eight days of high salinity (500 mM NaCl) treatment (Figure 2b).

Regarding carotenoid contents, moderate salinity caused significant reductions in *S. europaea* and *S. bigelovii* plants at the last sampling time, but not after one or three days of treatment with 300 mM NaCl. In contrast, under the same conditions, *S. persica* showed no significant differences in carotenoid concentrations compared to the control plants at any sampling time. However, significant reductions were observed in plants of the three *Salicornia* species after three or eight days of growth in the presence of 500 mM NaCl. The minimum carotenoid content (46% reduction compared to the non-stressed control) was observed in *S. europaea* three days after imposing high salinity treatment (Figure 2c). It should be noted that, in general, the *average* value of photosynthetic pigment levels in salt-treated plants was slightly higher in *S. persica* than in the other two species (Figure 2).

### 2.3. Cation Accumulation

**Figure 3:**
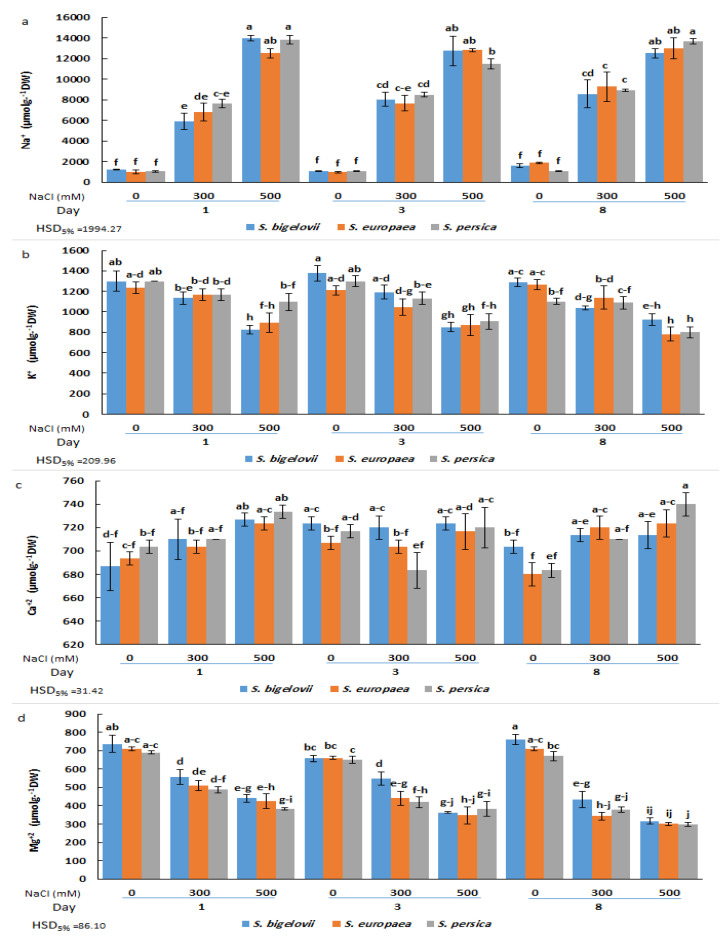
Effects of salt treatments (0, 300, and 500 mM NaCl) on sodium (Na^+^) (**a**), potassium (K^+^) (**b**), calcium (Ca^2+^) (**c**), and magnesium (Mg^2+^) (**d**) shoot contents of the three selected *Salicornia* species (*S. bigelovii*, *S. europaea*, and *S. persica*) at three sampling times (1, 3, and 8 days after starting the treatments). The values are means ± SD. Different lowercase letters over the bars indicate significant differences between mean values; HSD values (*p* ≤ 0.05) are included for comparing the means.

The analysis of variance showed a significant effect of the salt treatments on the concentrations of all analysed cations, Na^+^, K^+^, Ca^2+^, and Mg^2+^ (Table 1). There were also significant differences among sampling times for Na^+^, K^+^, and Mg^2+^ contents, whereas the effect of different *Salicornia* species was significant only for Mg^2+^ concentrations (Table 1). More importantly, the triple interaction effect of *Salicornia* species, salinity, and sampling time was significant on the concentrations of all analysed cations, Na^+^, K^+^, Ca^2+^, and Mg^2+^ (Table 1). Compared to non-saline conditions, a rapid and sharp increase (up to 13-fold) in the shoot Na^+^ concentration of *Salicornia* plants was observed in response to the salt treatments (Figure 3a). The results revealed that Na^+^ accumulation was dependent on the salt concentration of the irrigation solution, with levels significantly higher in plants subjected to high salinity than in those watered with 300 mM NaCl at all sampling times. At moderate salinity, mean Na^+^ contents showed an increasing trend with the duration of the treatment. Indeed, *S. persica* showed a rapid accumulation of Na^+^ ions after one day of moderate salinity treatment, with no further significant increase at later times. On the other hand, maximum Na^+^ contents in *S. europaea* and *S. bigelovii* plants were measured after eight days of treatment with 300 mM NaCl, values which were significantly higher than those of the first sampling time. In the presence of 500 mM NaCl, on the contrary, maximum Na^+^ levels were already measured after one day of treatment. No significant differences in Na^+^ contents were observed between the selected *Salicornia* species under any tested experimental condition (Figure 3a).

Salt stress reduced mean K^+^ concentrations (up to ca. 30%) in shoots of *Salicornia* plants in parallel to the increase in salinity. However, the differences with the control, non-stressed plants were statistically significant, generally only under high salinity conditions. An exception to this general pattern was the K^+^ content in *S. persica* after one day of treatment with 500 mM NaCl, which was not significantly different from the corresponding non-saline control or the moderate salinity treatment. The minimum K^+^ concentration was recorded for *S. persica* and *S. europaea* eight days after starting the severe salinity treatment. As for Na^+^, for each particular treatment and sampling time, no significant differences between species were generally detected (Figure 3b).

Compared to the other cations, Ca^2+^ concentration was less affected by salinity, showing small, generally non-significant fluctuations and a slightly increasing trend with increasing salinity, observed mainly after one and eight days of treatment. For example, a significant increase in shoot Ca^2+^ content was detected in *S. bigelovii* and *S. persica* after eight days of treatment with 500 mM NaCl, but amounting only to ca. 10% of the non-stressed controls (Figure 3c). On the contrary, a salinity- and time-dependent reduction of Mg^2+^ concentration was detected in all *Salicornia* species, with significant differences between severe salinity and non-saline conditions, but not between species. The minimum Mg^2+^ levels, about 40% of the corresponding controls, were measured eight days after exposure to high salinity (Figure 3d).

### 2.4. Shoot Osmolytes Contents

**Figure 4:**
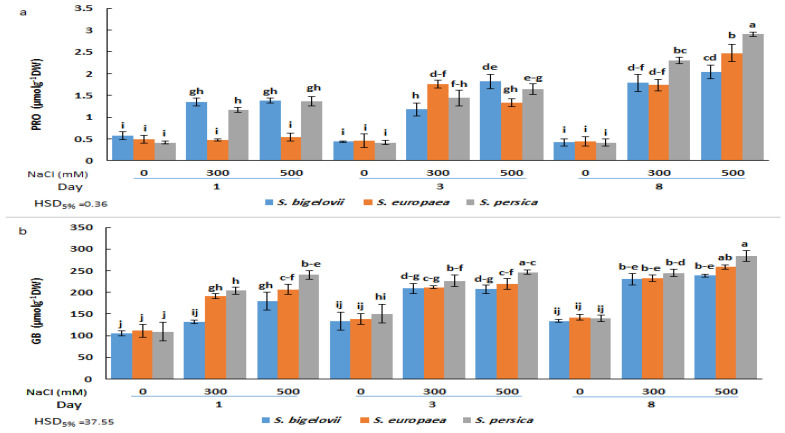
Effects of salt treatments (0, 300, and 500 mM NaCl) on proline (PRO) (**a**) and glycine betaine (GB) (**b**) shoot contents of the three selected *Salicornia* species (*S. bigelovii*, *S. europaea*, and *S. persica*) at three sampling times (1, 3, and 8 days after starting the treatments). The values are means ± SD. Different lowercase letters over the bars indicate significant differences between mean values; HSD values (*p* ≤ 0.05) are included for comparing the means.

The ANOVA results demonstrated that the effects of the main factors (species, salinity, and treatment time) and their interactions on proline and glycine betaine shoot concentrations were highly significant (Table 2). There were no significant differences in proline contents under non-saline conditions between the *Salicornia* species or sampling times. Proline concentrations augmented in response to the salt treatments in the three species but with different kinetics; in *S. bigelovii* and *S. persica*, significant increases were already observed one day after starting the treatments, whereas three days were required to observe the same response in *S. europea*. Maximum Pro levels were measured after eight days of severe salinity treatment, representing 4.8-, 5.6-, and 7-fold increases over non-stressed controls for *S. bigelovii*, *S. europea*, and *S. persica*, respectively. In this sampling, Pro contents in *S. persica* were significantly higher than in the other two species in the presence of 300 mM and 500 mM NaCl (Figure 4a). It should be noted that even the maximum Pro value reached in these experiments, 2.9 µmol g^−1^ DW, is probably too low to exert any substantial osmotic effect in the stressed plants. Nevertheless, proline may still contribute to salt stress tolerance through its additional functions as a low-molecular-weight chaperon, ROS scavenger, or signalling molecule [33].

The applied salt treatments also induced a significant accumulation of glycine betaine in a salinity- and time-dependent manner. For each salt concentration and sampling time, slight and, in most cases, non-significant differences in GB content were found between the three *Salicornia* species. However, *S. persica* generally showed higher mean GB amounts than *S. europaea* and *S. bigelovii*. The maximum relative increase in GB shoot concentration with respect to non-saline conditions was about 2-fold; however, it should be noted that background levels in the absence of salt were relatively high, over 100 µmol g^−1^ DW for the three *Salicornia* species (Figure 4b).

### 2.5. Oxidative Stress Markers

**Figure 5:**
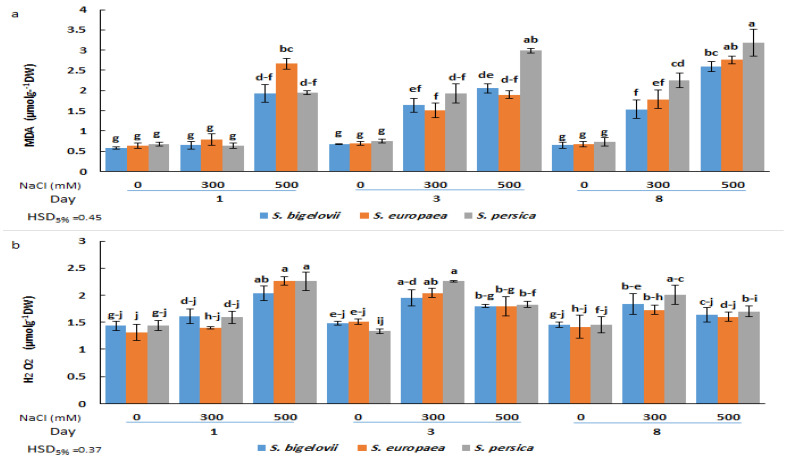
Effects of salt treatments (0, 300, and 500 mM NaCl) on malondialdehyde (MDA) (**a**) and hydrogen peroxide (H_2_O_2_) (**b**) shoot contents of the three selected *Salicornia* species (*S. bigelovii*, *S. europaea*, and *S. persica*) at three sampling times (1, 3, and 8 days after starting the treatments). The values are means ± SD. Different lowercase letters over the bars indicate significant differences between mean values; HSD values (*p* ≤ 0.05) are included for comparing the means.

Malondialdehyde (MDA) and hydrogen peroxide (H_2_O_2_) contents were determined as reliable indicators of oxidative damage caused by salt stress. The analysis of variance indicated significant effects of the factors studied, including *Salicornia* species, salinity, sampling times, and their interactions, on the accumulation of MDA and H_2_O_2_ (Table 2). Compared to the control plants grown in the absence of salt, MDA content increased significantly (up to 4.4-fold) in response to salinity treatments. This increase was already observed one day after subjecting the plants to severe salinity conditions; at later times, MDA also accumulated in plants watered with 300 mM NaCl. Under moderate- and high-salinity conditions, *S. persica* exhibited higher average levels of MDA accumulation than the other *Salicornia* species at three and eight days after exposure to salinity (Figure 5a). The maximum MDA content was recorded for *S. persica* eight days after the onset of high salinity treatment.

In plants subjected to high salinity (500 mM NaCl), shoot H_2_O_2_ contents rapidly increased in all *Salicornia* species, reaching maximum levels (up to 73% increase compared to the control) one day after starting the treatment, and then declined at later sampling times. In addition, a significant rise, compared to the control conditions, was found in H_2_O_2_ contents of all *Salicornia* species under moderate salinity at the second sampling time. No significant differences in H_2_O_2_ contents were found between the three investigated species under the same experimental conditions (Figure 5b).

### 2.6. Antioxidant Enzymes Activities

**Figure 6:**
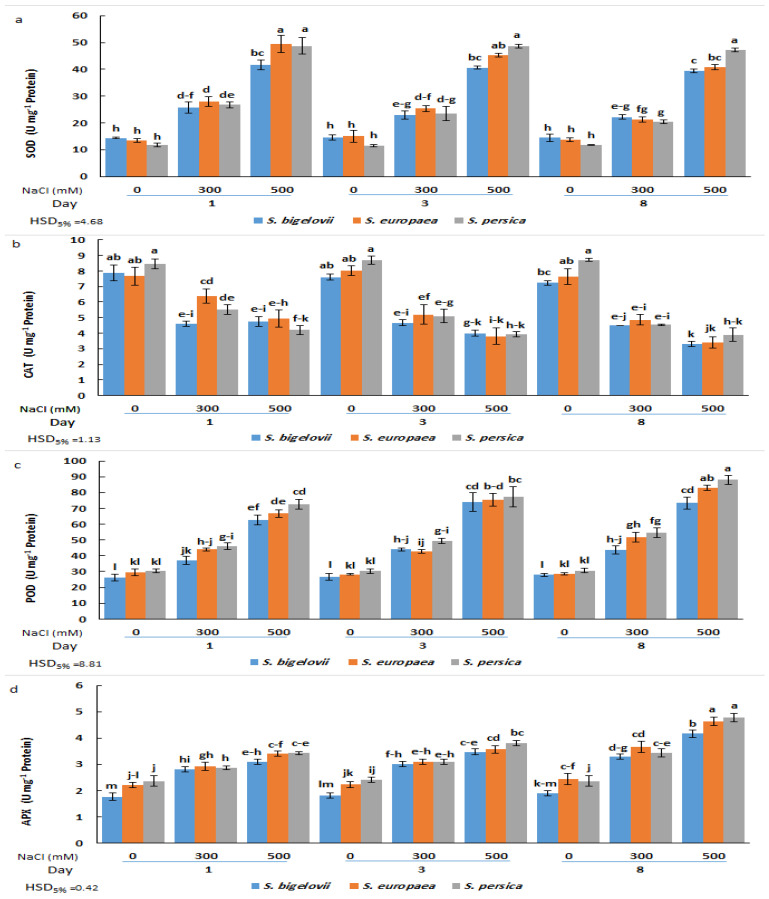
Effects of salt treatments (0, 300, and 500 mM NaCl) on the activities of superoxide dismutasa (SOD) (**a**), catalase (CAT) (**b**), peroxidase (POD) (**c**), and ascorbate peroxidase (**d**) in shoot extracts of the three selected *Salicornia* species (*S. bigelovii*, *S. europaea*, and *S. persica*) at three sampling times (1, 3, and 8 days after starting the treatments). The values are means ± SD. Different lowercase letters over the bars indicate significant differences between mean values; HSD values (*p* ≤ 0.05) are included for comparing the means.

The analysis of variance revealed that the activities of the antioxidant enzymes analysed (SOD, CAT, POD, and APX) were significantly affected by salinity levels, *Salicornia* species, and sampling times, as well as, in most cases, by their interactions (Table 2). SOD activity increased rapidly in the three species in response to the salt treatments; significant differences between high salinity, moderate salinity, and non-saline conditions were already observed after one day, and, for each treatment, the activities did not vary significantly at later sampling times. Comparing the tested *Saliconia* species, some statistically significant differences in SOD activity were found in plants treated with 500 mM NaCl, but not those grown in the absence of salt or subjected to moderate salinity; for example, *S. persica* plants showed significantly higher activity than those of *S. bigelovii* and *S. europaea* after eight days of treatment. *Salicornia persica* was also the species with the maximum salt-induced increase in SOD activity, showing a more than 4-fold increase over control values at all sampling times (Figure 6a).

Variations in CAT activity showed an opposite pattern than that of SOD, with maximum mean values calculated under non-saline conditions and a general decreasing trend parallel to the increase in NaCl concentration and the duration of the treatments. However, the differences between moderate and high salinity or between samplings were not statistically significant in some cases. Also, with few exceptions, no significant differences existed between species for plants subjected to the same treatments (Figure 6b).

As for SOD, POD and APX activities were induced by salt stress. POD activity progressively increased with increasing salinity levels and sampling times, and the maximum POD activity was observed in *S. persica* and *S. europaea* eight days after exposure to 500 mM NaCl, representing in both cases a relative increase of 2.9-fold over the activity determined in non-stressed plants. Here again, for each specific treatment, differences between species were generally non-significant (Figure 6c). Changes in APX activity followed the same pattern as POD; that is, a general ascending trend in response to salinity treatments and sampling time, although the differences with the non-stressed controls were relatively weaker. Thus, the maximum APX activity levels, measured in plants grown for eight days in the presence of 500 mM NaCl, represented an increase of about twofold with respect to the corresponding controls for the three *Salicornia* species (Figure 6d). Therefore, except for CAT, the tested antioxidant activities increased in response to the salt treatments, reaching slightly higher levels in *S. persica* than in *S. europaea* or *S. bigelovii* plants (Figure 6).

### 2.7. Correlation Analysis

**Figure 7:**
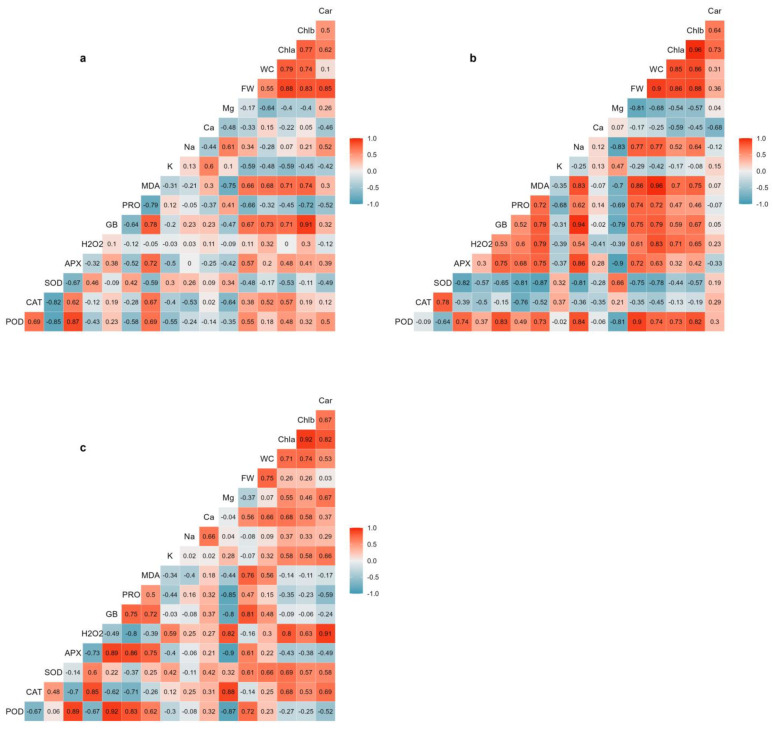
Pearson’s correlation coefficients between the different traits determined under non-saline conditions (**a**), moderate-salinity (300 mM NaCl) (**b**), and high-salinity (500 mM NaCl) (**c**) conditions. The colour gradient shows correlation ranges from blue (positive) to red (negative). Abbreviations: carotenoids (Car); chlorophyll b (Chlb); chlorophyll a (Chla); water content (WC); fresh weight (FW); magnesium (Mg); calcium (Ca); sodium (Na); potassium (K); malondialdehyde (MDA); proline (PRO); glycine betaine (GB); hydrogen peroxide (H_2_O_2_); ascorbate peroxidase (APX); superoxide dismutase (SOD); catalase (CAT); peroxidase (POD).

The correlation coefficients between the analysed variables were calculated for each salt treatment (Figure 7). The results indicated that the interrelationships between the different traits were affected by the salinity conditions. The strongest positive correlation between plant biomass (shoot FW) and water content was obtained under moderate salinity conditions (300 mM NaCl), which are optimal for *Salicornia* plant growth. Water content was also highly positively correlated with MDA, chlorophylls a and b, H_2_O_2_, glycine betaine, Na^+^, POD activity, and proline under moderate salinity conditions. Substantial correlations were also found between water content and chlorophylls a and b, Ca^2+^, and SOD activity under high salinity conditions. There were significant positive correlations between plant biomass and the contents of chlorophylls a and b under non-saline and moderate salinity conditions (Figure 7a,c), although plant biomass was not correlated with photosynthetic pigments under high salinity conditions. While no strong and significant correlations were found between plant biomass and cations concentrations under non-saline and high-salinity conditions (Figure 7a,c), a strong positive correlation was determined between Na^+^ and plant biomass under moderate salinity (Figure 7b). Moreover, Mg^2+^ concentration was negatively and significantly correlated with plant biomass under moderate salinity (Figure 7b).

The results also revealed that the osmolytes, glycine betaine and proline, significantly correlated with plant biomass. The relationship between glycine betaine and plant biomass was less affected by salinity treatments, as glycine betaine content was highly and positively correlated with plant biomass under all tested conditions (Figure 7). Proline content, on the other hand, displayed a relatively strong negative correlation with shoot FW in non-stressed controls (Figure 7a) but a positive correlation in moderate salinity-treated plants (Figure 7b). The oxidative damage indicators, MDA and H_2_O_2_, exhibited different correlation patterns with plant biomass. Strong positive correlations existed between MDA content and plant biomass under moderate (Figure 7b) and high (Figure 7c) salinity conditions. On the other hand, H_2_O_2_ content was positively correlated with plant biomass only under moderate-salinity levels (Figure 7c). H_2_O_2_ was also strongly negatively correlated with APX and POD activities under high salinity (Figure 7c). The antioxidant enzymes showed different patterns of correlations with plant biomass under saline conditions. APX and POD activities were positively and significantly correlated with plant biomass under moderate-salinity conditions (Figure 7b), whereas there was a strong negative correlation between SOD and plant biomass under such conditions. In addition, POD followed by APX and SOD displayed considerable positive correlations with plant biomass under high salinity conditions. Indeed, APX and POD activities correlated consistently with each other, as shown by significant positive correlation coefficients, under all the conditions tested (Figure 7). No meaningful correlations were found between CAT activity and *Salicornia* growth parameters (Figure 7).

### 2.8. Principal Component Analysis

**Figure 8:**
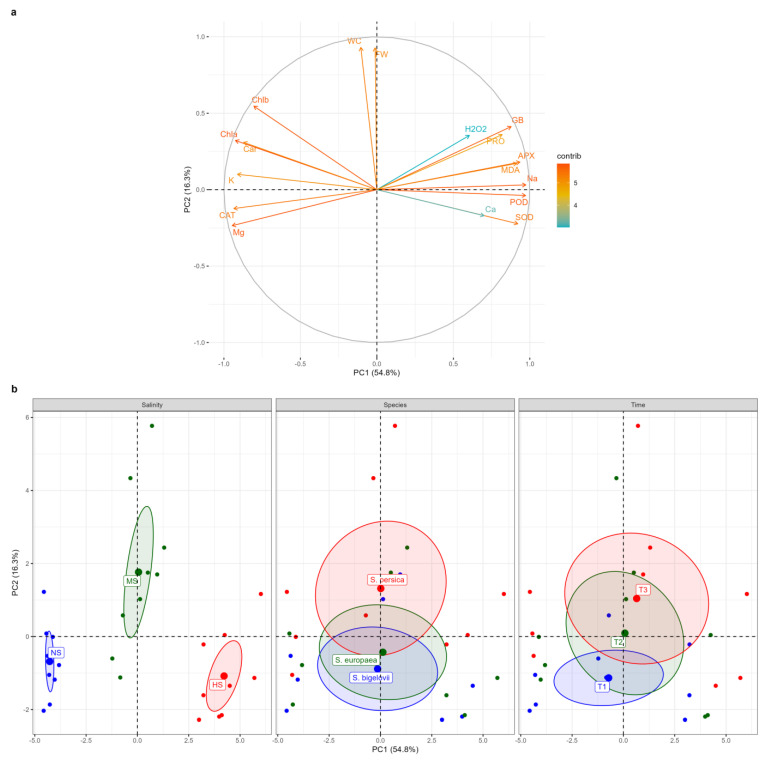
Principal component analysis (PCA). Loading plot of the first two principal components for the variables examined (**a**). Abbreviations: fresh weight (FW); water content (WC); hydrogen peroxide (H_2_O_2_); malondialdehyde (MDA); glycine betaine (GB); proline (PRO); peroxidase (POD); catalase (CAT); superoxide dismutase (SOD); ascorbate peroxidase (APX); sodium (Na^+^); potassium (K^+^); calcium (Ca^2+^); magnesium (Mg^2+^). PCA score plots (**b**) for salt treatments: non-saline (NS), moderate salinity (MS), and high salinity (HS); *Salicornia* species; *S. bigelovii*, *S. europaea*, and *S. persica*; and sampling times: one day (T1), three days (T2), and eight days (T3) after starting the treatments.

Principal component analysis (PCA) was performed to further assess the relationships amongst the measured parameters and to determine and visualise the magnitude and direction of correlations between the variables and the extracted principal components (PCs) (Figure 8). The first two PCs explained over 71% (54.8% and 16.3%, respectively) of the total variation (the eigenvectors are reported in Supplementary Table S1). The correlation circle and the biplot of the first two components, PC1 and PC2, are represented in Figure 8. The salinity levels were well separated on PC1, which accounted for more than 54% of the total variation. The high-salinity treatment (500 mM NaCl) was located on the positive side of PC1, whereas the non-saline treatment fell onto the negative side. The results revealed strong positive contributions of APX, POD, Na^+^, glycine betaine, SOD, MDA, and proline to PC1, associated with the data cluster of high-salinity conditions, indicating that Na^+^ accumulation was associated with the enhanced activities of the antioxidant enzymes and the increased contents of the osmolytes. Conversely, Mg^2+^, CAT, K^+^, and photosynthetic pigments were strong negative contributors to PC1, associated with the data cluster of non-saline conditions. Moreover, fresh weight and water content of *Salicornia* plant shoots were strongly positively correlated with PC2, which explained more than 16% of the total variation (Figure 8a). Plotting data based on PC1 and PC2 confirmed that *Salicornia* plants exposed to moderate salinity conditions had higher fresh shoot weight and water content percentage. PC2 separated the data according to the sampling times, as the data from one-day sampling were positioned on the negative side of PC2, whereas the data from the eight-day sampling were distributed on the positive side of PC2, tending to correspond to plant biomass under moderate-salinity conditions (Figure 8b). The biplot showed that the barycenters of *S. europaea* and *S. bigelovii* were close to each other, implying that they reacted similarly to salt stress, but *S. persica* data were partially separated from the other species, shifting toward the positive side of PC2, in which its barycenter corresponded to that of moderate-salinity conditions. As a result, *S. persica* exhibited a relatively higher salinity tolerance than the other two *Salicornia* species under moderate-salinity conditions (Figure 8b).

### 2.9. Hierarchical Clustering

**Figure 9:**
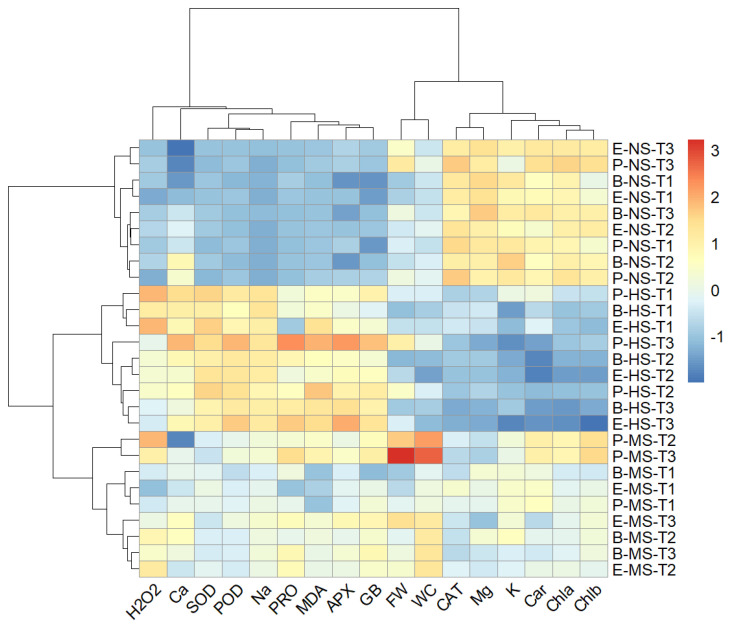
Two-dimensional hierarchical clustering heat map of the traits measured in shoots of the three *Salicornia* species, *S. europaea* (E), *S. persica* (P), and *S. bigelovii* (B) under non-saline (NS), moderately saline (MS), and highly saline (HS) conditions, and after one day (T1), three days (T2), and eight days (T3) of applying the salt treatments. Abbreviations: fresh weight (FW); water content (WC); hydrogen peroxide (H_2_O_2_); malondialdehyde (MDA); glycine betaine (GB); proline (PRO); peroxidase (POD); catalase (CAT); superoxide dismutase (SOD); ascorbate peroxidase (APX); sodium (Na^+^); potassium (K^+^); calcium (Ca^2+^); magnesium (Mg^2+^).

A two-way hierarchical cluster analysis was performed to discover groups of similar variables and groups of similar treatments. A heat map generated using two-way cluster analysis is shown in Figure 9. The clustering results were consistent with the results from the PCA and confirmed that salinity level was the major discriminating factor. Accordingly, irrespective of the *Salicornia* species and sampling times, the data measured in non-saline, moderate-, and high-salinity treatments formed distinct clusters. Moreover, the data collected one day after starting the salt treatments on *Salicornia* plants were well separated from those measured at later sampling times (Figure 9). Clustering the biochemical and growth parameters revealed a distinct group, including proline, glycine betaine, Na^+^, Ca^2+^ MDA, H_2_O_2_, SOD, APX, and POD, which generally showed maximum contents or activities under high salinity conditions (Figure 9). In addition, the fresh weight and water content of *Salicornia* shoots were grouped together and separated from a cluster containing chlorophylls a and b, carotenoids, K^+^, Mg^2+^, and CAT, which, contrary to the other traits, showed their minimum values in the presence of salt (Figure 9).

## 3. Discussion

*Salicornia* species utilise several cellular adaptive mechanisms that allow them to grow in saline habitats. Enhancing our understanding of the salt-induced biochemical responses is essential to decipher further how *Salicornia* plants adapt to high salinity. Moreover, the analysis of temporal changes in short-term biochemical responses to salinity of *Salicornia* species may provide insights into the dynamics of these responses, identifying early indicators of salinity stress, and, finally, an in-depth understanding of adaptation mechanisms. The present study evaluated changes in biomass and biochemical parameters in plants of three *Salicornia* species during exposure to varying NaCl concentrations for different times. The shoot biomass of *Salicornia* plants was measured as a distinctive morphological indicator of plant growth performance [31]. *Salicornia* plants showed optimum growth when exposed to moderate salinity (300 mM NaCl), indicating their capability to counter osmotic, oxidative, and ionic stresses. The increased growth and water content of *Salicornia* shoots at moderate salinity may result from the accumulation of organic solutes and essential ions to maintain cell turgor pressure, eventually making plants more succulent. In accordance with these results, previous studies have demonstrated a positive effect of a certain amount of NaCl on the growth of different *Salicornia* species [16,18,19,20,28,30,31]. However, the optimum salinity levels reported varied between 100 and 400 mM NaCl, possibly due to interspecific genetic variation for salinity tolerance as well as different times of measuring plant biomass after salt stress application. Throughout the experiment, we measured plant biomass three times, which helped clarify growth differences between *Salicornia* species in response to varying salinity levels. It is noteworthy that *S. persica* could maintain its growth under high salinity conditions (500 mM NaCl) and produced significantly higher amounts of shoot biomass than *S. europaea* and *S. bigelovii*, indicating a higher salinity tolerance in *S. persica.* This finding agreed with a previous study that reported higher salinity tolerance in *S. persica* than in *S. europaea* [16].

Our results demonstrated that photosynthetic pigment contents (chlorophylls and carotenoids) were negatively affected by salinity, particularly at higher NaCl concentrations, in the three investigated *Salicornia* species. Salt-induced reductions in photosynthetic pigments have been previously observed in different *Salicornia* species [19,21,22]. The loss of chlorophylls and carotenoids in response to salt stress may be due to the inactivation of enzymes associated with their biosynthesis or excessive ROS generation [34]. However, non-significant variations in the contents of photosynthetic pigments were generally observed in *S. persica* between non-saline and moderate salinity conditions over the study’s time course, implying that this *Salicornia* species can maintain these parameters, to a certain extent, in moderately saline environments. Consequently, the relatively higher concentrations of photosynthetic pigments may contribute to the better growth of *S. persica* compared to the other *Salicornia* species under moderate-salinity conditions. The lack of correlation between growth parameters and photosynthetic pigments under high-salinity treatment suggested that the growth performance of *Salicornia* species under such conditions may not be explained by variations of photosynthetic pigments, contrary to what was reported in the halophytes *Arthrocnemum macrostachyum* and *Sarcocornia fruticosa* [20].

Many halophytic species can efficiently transport Na^+^ to the shoots and compartmentalise it into vacuoles to regulate osmotic potential and maintain water uptake [35]. Accordingly, our results revealed a significant sodium accumulation in the shoots of the three *Salicornia* species under moderate and high salinity. Similar results have been reported in different species of this genus [16,18,21,28]. Furthermore, rapid and sharp increases in shoot Na^+^ contents in *Salicornia* plants under moderate- and high-salinity conditions, shown by temporal measurements, indicated that *Salicornia* species utilise sodium transport to the shoots as an early response mechanism to deal with salt stress. Previous studies have also reported short-term rapid sodium accumulation in response to salt stress [36,37,38]. In addition to the significant contribution of sodium to *Salicornia* adaptive responses to salinity, shown with the PCA, it should be noted that sodium content had strong positive correlations with osmolyte (proline and glycine betaine) concentrations under moderate salinity. These findings indicated that the studied *Salicornia* species could effectively re-establish osmotic and ion homeostasis under moderate salinity, resulting in maximum plant growth.

The *Salicornia* species exhibited a slight, and in most cases non-significant, decrease in shoot K^+^ contents in response to moderate salinity, with little temporal variation. Potassium retention is considered a vital component of salinity tolerance in halophytes, as K^+^ plays key roles in signal transduction and osmotic adjustment [39]. Several studies reported slight changes in K^+^ contents in halophytes in response to salt stress [20,40,41]. In fact, the relative retention of K^+^ under moderate salinity would not have been appropriately detected without temporal sampling. On the other hand, a significant decline in K^+^ concentrations was observed in the high salinity (500 mM NaCl) treatment, which was likely due to the antagonism between Na^+^ and K^+^ in their response to salt stress, confirmed with the PCA. Actually, a decrease of K^+^ in response to increasing Na^+^ concentrations represents the expected pattern of variation as both cations, which have similar physicochemical properties, compete for the same binding sites in proteins, including K^+^ membrane channels and transporters [42,43].

The salinity treatments generally caused a slight increase in the shoot Ca^2+^ contents of the *Salicornia* species. It has been previously reported that *Salicornia* plants can retain calcium under moderate- and high-salinity conditions [20,21]. Furthermore, the correlation analysis revealed that Ca^2+^ and Na^+^ concentrations were positively associated under high salinity conditions, implying that Ca^2+^ contributes to salinity tolerance in *Salicornia*. Ca^2+^ acts as a critical component in signalling pathways regulating responses of the whole plant to salinity [44]. Our results also uncovered that Mg^2+^ temporally decreased in response to moderate and high salt-stress treatments, which agrees with previous studies on *Salicornia herbacea* [30] and *Salicornia rubra* [45]. The adverse effect of high salinity on plant growth may be partly related to a deficiency of essential nutrients, such as Mg^2+^ [46].

Proline and glycine betaine are compatible osmolytes that generally accumulate in response to osmotic stress due to drought and high salinity [47]. The present study revealed that proline and glycine betaine levels were significantly and progressively enhanced in plants of the three *Salicornia* species subjected to moderate- and high-salinity treatments. Furthermore, multivariate statistical analyses illustrated the significant contributions of these osmolytes to the adaptive responses of *Salicornia* plants to salinity. Increased proline concentrations in response to moderate and high salinity levels have been reported in *S. europaea*, *S. persica*, and *S. prostrata* [16,20,27,28,48]; however, Parida and Jha [18] found proline accumulation in *Salicornia brachiata* only under high salinity (600 mM NaCl). Proline is the most common compatible solute in plants [33]. Proline accumulation generally maintains osmotic homeostasis and protects enzymes and membranes from ion toxicity under salt stress [49]. Nevertheless, it is noteworthy that *Salicornia* plants exhibited very low amounts of proline in the shoots compared to glycine betaine. This finding is in agreement with previous studies, which found high glycine betaine and low proline contents in some other *Salicornia* species [21,27] or in the related halophyte *Sarcocornia fruticosa* [50], implying that glycine betaine is a pivotal osmolyte contributing to salinity tolerance in these halophytic taxa. Moreover, high glycine betaine levels were also observed under non-saline conditions, suggesting that this osmolyte confers a partially constitutive defence mechanism to *Salicornia* species against salt-induced osmotic stress. This result aligns with those reported by Calone et al. [21] for some other *Salicornia* species. Also, in a field study including plants of the related species *Sarcocornia fruticosa*, shoot glycine betaine levels were maintained high and practically constant throughout the year despite the drastic seasonal changes observed in soil salinity and soil Na^+^ and Cl^−^ concentrations [50]. In halophytes, glycine betaine is present at much higher levels than in glycophytes [51]. Furthermore, glycine betaine rapidly accumulated in response to the salt treatments, although further gradual increases occurred at later times. As a result, glycine betaine can be considered an early response indicator for salt stress. Glycine betaine not only acts as an osmolyte, but also interacts with both hydrophilic and hydrophobic domains of protein complexes, stabilising and protecting these molecules from ROS deleterious effects [51]. Several proline and glycine betaine measurements during the experiment helped to interpret the results more accurately.

The contents of oxidative stress markers, MDA and H_2_O_2_, were determined to assess the extent of oxidative stress on *Salicornia* plants exposed to salt treatments. The content of MDA, an end product of lipid peroxidation [52], reflects the degree of cell membrane damage due to ROS accumulation in plants subjected to environmental stresses. Shoot MDA concentrations increased in *Salicornia* plants in response to salt stress and showed a positive correlation with *Salicornia* growth under high-salinity conditions. Similarly, increased levels of MDA have been reported in response to salt treatments in *S. persica* [16,53], *S. europaea* [16], *S. brachiata* [18], and other halophytes, including *Arthrocnemum macrostachyum* and *Sarcocornia fruticosa* [20]. In addition, a positive relationship between MDA and the growth of *Salicornia* plants subjected to salinity treatments has been previously documented [17,18,53], suggesting the plant’s ability to tolerate oxidative stress or exploit salt as a signalling molecule for growth. However, it should be noted that this relationship is complex, and may vary depending on the plant species and the salinity level in its environment. *Salicornia* plants showed a significant elevation in H_2_O_2_ contents over the non-saline conditions up to the first three days after exposure to moderate and high salinity levels; this response may be due to an impairment of H_2_O_2_ scavenging, imposed by oxidative stress at later times. Increased contents of H_2_O_2_ in response to high salinity have been previously found in *S. europaea* [16,19]. However, analysis of temporal changes of H_2_O_2_ contents showed no further increase and remained relatively stable at the last sampling time (after eight days of applying the salt treatment), in parallel to the increased activities of antioxidant enzymes such as POD and APX, which catalyse its elimination. This finding suggested that these halophytes possess effective antioxidant systems to cope with oxidative stress by preventing the overproduction of H_2_O_2_. H_2_O_2_ is an essential molecular signal mediating several biological processes responding to environmental stresses [54].

One of the harmful effects of salinity on plant growth and development is the excessive generation of ROS, such as superoxide radicals, hydrogen peroxide, and hydroxyl radicals. Halophytes are supposed to activate efficient antioxidant mechanisms to remove ROS and mitigate oxidative damage under saline conditions [55]. Hence, we evaluated the activities of four antioxidant enzymes (SOD, CAT, POD, and APX) to better assess *Salicornia*’s adaptive responses. The *Salicornia* species exhibited a rapid and significant induction of all antioxidant activities, except CAT, in response to moderate and high salinity treatments. SOD, POD, and APX generally showed similar temporal trends, reaching the maximum activities eight days after starting the moderate and high salinity treatments. Similarly, SOD and APX activities have been reported to temporally increase in *S. brachiata* plants subjected to different salinity levels [18]. SOD enzymes constitute the protection front line against ROS by dismutating superoxide radicals into hydrogen peroxide and oxygen [56]. Previous studies also reported enhanced levels of SOD activities upon different salt treatments in *S. persica* [16,53], *S. europaea* [16], and some other halophytes such as *Suaeda maritima* [57] and *Zygophyllum coccenium* [58]. It is worth mentioning that *S. persica* could maintain a high level of SOD activity under high salinity conditions at all sampling times, which probably contributes to the higher salinity tolerance of *S. persica* compared to the other two species. Accordingly, a higher increase in *S. persica* SOD activity has been previously associated with its higher salinity tolerance compared to *S. europaea* [16].

Opposite to SOD, the *Salicornia* CAT activities decreased with increasing salinity and treatment times. This agrees with the results reported by Parida and Jha [18], who demonstrated a decline in CAT activities of *S. brachiata* with extended exposure to moderate and high salinity. This variation pattern suggests that CAT is a less efficient scavenger of H_2_O_2_ in *Salicornia*, which could be explained by its relatively poor affinity for H_2_O_2_ and its photo-inactivation with subsequent degradation in the presence of light [59].

APX and POD activities generally increased in shoots of the *Salicornia* plants in response to increased salinity levels and sampling times; therefore, the highest APX and POD activities were determined eight days after starting the treatment with 500 mM NaCl. Furthermore, the significant contributions of APX and POD to *Salicornia* growth under salt stress are supported by the applied statistical analyses, including the determination of correlation coefficients, PCA, and cluster analysis. To our knowledge, no previous reports have discussed the crucial role of APX in response to salinity in the three *Salicornia* species investigated here, although a salt-induced increase in APX activity has been observed in *S. brachiata* [18] and other halophytes [60,61]. Also, in agreement with our data, enhanced POD activity under salt stress conditions has been documented in *S. europaea* [19,20]. Therefore, the present study supports the notion that APX and POD play major protective roles against oxidative stress, contributing toward salinity tolerance in *Salicornia* species.

## 4. Materials and Methods

### 4.1. Plant Material

The study was conducted in the greenhouse of the Department of Plant Production and Genetics, School of Agriculture, Shiraz University, Shiraz, Iran. The experiment was performed in a factorial arrangement based on a completely randomised design with three replicates to assess the effects of three salinity levels (0, 300, and 500 mM NaCl) on biochemical traits of three *Salicornia* species (*S. persica*, *S. europaea*, and *S. bigelovii*), at three sampling times (1, 3, and 8 days after starting the treatments). *Salicornia* seeds were obtained from the Seed and Plant Certification and Registration Institute of Iran. Seeds were sown in plastic pots (diameter: 21 cm; height: 20 cm) filled with 4.5 kg of a sterilised mixture of clay loam soil: sand: perlite (2:1:1). The soil mixture had a field capacity (FC) of 14%, a pH of 7.2, and an electrical conductivity (EC) of 0.8 dS/m. Considering the need for multiple samples, four pots were assigned to each of nine combinations of salinity levels and *Salicornia* species per replicate. The pots were regularly irrigated with tap water (EC = 0.5 dS/m) to maintain soil moisture at the level of FC. Two-month-old plants of uniform size were subjected to salt treatments (irrigation with 300 mM and 500 mM NaCl aqueous solutions). The plants were grown under greenhouse conditions with natural photoperiod, average day/night temperatures of 25 °C/20 °C, and relative humidity of 70% ± 5%. Shoots were collected at the established sampling times from plants of each *Salicornia* species subjected to salt treatments or grown in the absence of salt (controls).

### 4.2. Growth Parameters

The growth of *Salicornia* plants was evaluated based on the changes in fresh and dry weights of plant shoots in response to the treatments applied. Four plants from each species per replicate were harvested at 1, 3, and 8 days after starting the treatments, and the fresh weight of the shoots was immediately determined. Part of the shoot material was then placed into an oven at 70 °C for 48 h and weighed again to record dry weights. The water content percentage of each sample was determined according to the following formula:

WC (%) = [(FW − DW)/FW] × 100

### 4.3. Photosynthetic Pigments

The contents of photosynthetic pigments, including chlorophyll a (Chl. a), chlorophyll b (Chl. b), and carotenoids (Car), were spectrophotometrically determined according to a previously described protocol [62]. Fresh plant shoots (0.1 g) were homogenised in 10 mL of 80% acetone in the dark until the residue became colourless, and then centrifuged at 13,000× *g* for 10 min. The supernatant absorbance was read at 470, 646, and 663 nm, and the contents of chlorophylls and carotenoids were calculated using the equations described by Lichtenthaler and Wellburn [62].

### 4.4. Quantification of Cations

Shoot samples were oven-dried at 70 °C for 48 h and finely ground to quantify cations (Na^+^, K^+^, Ca^2+^ and Mg^2+^). The samples (0.5 g each) were reduced to ashes at 580 °C for 4 h and then digested with 5 mL of 2 N HCl. The resulting solutions were passed through filter paper, and the samples were diluted with double distilled water to a final volume of 50 mL. Na^+^ and K^+^ were quantified with a flame photometer (PFP7, Jenway, Staffordshire, UK) [63], whereas Ca^2+^ and Mg^2+^ were determined using an atomic absorption spectrometer (AA-670, Shimadzu, Kyoto, Japan) [64].

### 4.5. Proline and Glycine Betaine

The method of [65] was used to measure proline content. For each sample, 0.5 g of plant shoot was homogenised in 10 mL of 3% (*w*/*v*) sulphosalicylic acid. The mixture was then filtered through Whatman No. 2 filter paper. The extract was mixed with acid ninhydrin reagent and acetic acid, incubated at 100 °C for 1 h, and then cooled on ice for 10 min. The sample was extracted with toluene, and the organic phase was used to determine spectrophotometrically proline content by reading the absorbance at 520 nm, with toluene as the blank, in a UV-visible spectrophotometer (7315 UV/VIS, Jenway, Staffordshire, UK). Proline concentrations, expressed in µmol g^−1^ DW, were calculated from a standard curve obtained using parallel assays with known proline concentrations.

Glycine betaine contents were determined following the method described by Grieve and Grattan [66]. Dried and finely ground shoot material (0.5 g) was shaken with 20 mL of distilled water for 24 h at 25 °C. Following filtration, the extract was diluted (1:1) with 2 N H_2_SO_4_ and cooled in ice for 1 h. Then, 0.5 mL of the sample was added to 0.2 mL of cold KI-I_2_ reagent and gently vortexed. The mixture was kept at 4 °C for 16 h and subsequently centrifuged at 10,000× *g* for 15 min at 0 °C. The supernatant was removed, and the formed crystals were dissolved into 9 mL of cold 1,2- dichloroethane. After two hours, the absorbance was recorded at 365 nm. Glycine betaine concentration was estimated using a standard curve and expressed as μmol g^−1^ DW.

### 4.6. MDA and H_2_O_2_

Lipid peroxidation of the samples was estimated by measuring MDA content [67]. For each plant sample, 0.25 g of shoots were homogenised in 5 mL of 0.1% trichloroacetic acid (TCA) and centrifuged at 10,000× *g* for 5 min at 4 °C. An amount of 1 mL of the supernatant was mixed with 4 mL of 0.5% (*w*/*v*) thiobarbituric acid (TBA) in TCA (20% *w*/*v*). The mixture was heated at 95 °C for 30 min, immediately cooled on ice, and then centrifuged at 10,000× *g* for 10 min. The absorbance of the supernatant was measured at 532 nm and 600 nm (non-specific turbidity). MDA content was calculated from the difference between the absorbance values using an extinction coefficient of 155 mM^−1^ cm^−1^.

Hydrogen peroxide content was quantified using the method of [68]. In total, 0.5 g of fresh plant shoots was homogenised in an ice bath with 5 mL of 0.1% TCA. After centrifugation at 12,000× *g* for 15 min, 0.5 mL of the supernatant was added to 0.5 mL of 10 mM potassium phosphate buffer (pH 7.0) and 1 mL of 1 M potassium iodide. The absorbance was read at 390 nm, and the H_2_O_2_ content was calculated from a standard curve and expressed as μmol g^−1^ DW.

### 4.7. Antioxidant Enzyme Assays

Plant shoot samples (0.5 g) were homogenised in ice-cold phosphate buffer (pH 7.6). The homogenates were centrifuged at 13,000× *g* for 15 min at 4 °C, and the antioxidant enzyme activities were measured spectrophotometrically in the supernatants. Protein contents in the supernatants were measured according to Bradford’s method [69]. SOD activity was determined based on the inhibition of the photochemical reduction of nitro blue tetrazolium (NBT) by superoxide radicals [70]. One SOD unit was defined as the amount of enzyme required for 50% inhibition of NBT reduction at 560 nm. CAT activity was estimated by measuring at 240 nm the rate of disappearance of H_2_O_2_ [71]. One CAT unit was defined as the amount of enzyme required to decompose one mmol H_2_O_2_ per minute under the assay conditions. POD activity was assayed following a previously published protocol [72]. The measurement is based on monitoring guaiacol oxidation at 470 nm in the presence of H_2_O_2_. The absorbance of the reaction solution was read for 1 minute at 10-s intervals. One unit of POD activity was defined as the amount of enzyme causing an absorbance change of 0.01 per minute. APX activity was measured based on the decrease of the absorbance at 290 nm due to the oxidation of ascorbic acid [73]. One unit of APX activity was defined as the amount of enzyme oxidising one mmol ascorbate per minute.

### 4.8. Statistical Analyses

Analysis of variance (ANOVA) was performed to test the effects of salinity, *Salicornia* species, sampling times, and their interactions on the measured variables. Mean comparisons were conducted using Tukey’s HSD test at *p* ≤ 0.05. Pearson’s correlation coefficients, principal component analysis (PCA), and hierarchical cluster analysis using Ward’s method were conducted to establish the relationships between the analysed traits and to explore their relative significance on *Salicornia* growth under salinity conditions. R statistical software (Version 4.3.2; https://www.R-project.org; accessed on 20 November 2023) was used for all statistical analyses.

## 5. Conclusions

This study clearly shows changes in shoot biomass and several biochemical parameters of the three selected *Salicornia* species in response to moderate- and high-salinity treatments in a temporal sequence. The maximum growth of *Salicornia* plants was observed under moderate salinity. The results presented here identified the biochemical attributes with vital contributions to salinity tolerance of *Salicornia* through the maintenance of osmotic and ion homeostasis and protection against oxidative stress. *Salicornia* plants activate an efficient antioxidant enzymatic system that mainly relies on APX and POD activities to alleviate oxidative stress under moderate salinity conditions. The results also reveal that glycine betaine accumulation, both constitutive and induced by salt stress, represents a critical tolerance mechanism through osmotic adjustment in *Salicornia* plants subjected to salinity. Furthermore, high and rapid Na^+^ accumulation coupled with a relative retention of K^+^ and Ca^2+^ in plants subjected to salt stress treatments is another significant biochemical response contributing to osmotic and ionic balance, conferring salinity tolerance to *Salicornia* species. Notably, the relative retention of K^+^ and Ca^2+^ under moderate salinity could not be accurately inferred without measurements at different treatment times. We also showed that although the three investigated *Salicornia* species use the same biochemical mechanisms to respond to salt stress, *S. persica* is more tolerant to salinity than *S. europaea* and *S. bigelovii*, as evidenced by its better growth under moderate and high salinity conditions. This higher salinity tolerance was associated with higher activities of antioxidant enzymes, SOD, POD and APX, and relatively higher accumulation of glycine betaine and proline in *S. persica* compared to the other *Salicornia* species. Overall, this study provided relevant information on the role and significance of different biochemical response mechanisms on salinity tolerance in *Salicornia* species.

## Figures and Tables

**Table 1:** Analysis of variance (ANOVA) for the variables measured in three *Salicornia* species (*S. persica*, *S. europea*, and *S. bigelovii*) under non-saline, moderate (300 mM NaCl), and high (500 mM NaCl) salinity conditions at three sampling times (1, 3, and 8 days after starting the treatments). Abbreviations: fresh weight (FW); water content (WC); chlorophyll a (Chl. a); chlorophyll b (Chl. b); carotenoids (Car). sodium (Na^+^); potassium (K^+^); calcium (Ca^2+^); magnesium (Mg^2+^).

				Mean Squares						
	Df	FW	WC	Chl. a	Chl. b	Car	Na^+^	K^+^	Ca^2+^	Mg^2+^
Species	2	0.88 **	17.83 **	1.26 **	0.87 **	0.24 **	218,549 ^ns^	10,357 ^ns^	211 ^ns^	19,505 **
Salinity	2	0.40 **	135.49 **	20.89 **	8.13 **	2.28 **	935,837,253 **	996,868 **	4293 **	787,916 **
Time	2	0.79 **	14.86 **	0.02 ^ns^	0.31 **	0.16 **	4,431,883 **	40,298 **	70 ^ns^	46,001 **
Species × Salinity	4	0.05 *	2.72 *	0.10 ^ns^	0.19 **	0.03 ^ns^	857,253 ^ns^	15,407 *	393 **	3842 **
Species × Time	4	0.07 **	2.98 *	0.10 ^ns^	0.06 ^ns^	0.01 ^ns^	882,716 ^ns^	29,642 **	331 *	777 ^ns^
Salinity × Time	4	0.10 **	14.64 **	0.49 **	0.42 **	0.19 **	4,675,309 **	4098 ^ns^	1096 **	16,638 **
Species × Salinity × Time	8	0.02 *	0.62 ^ns^	0.03 ^ns^	0.04 ^ns^	0.02 *	1,188,642 **	15,534 **	257 *	1948 **
Residuals	54	0.008	1.11	0.07	0.05	0.01	392,747	4352	98	732
CV%		4.11	1.33	10.48	10.50	9.87	8.51	6.05	1.38	5.36

^ns^: Non-significant; *: Significant at *p* < 0.05; **: Significant at *p* < 0.01.

**Table 2:** Analysis of variance (ANOVA) for the biochemical variables measured in three *Salicornia* species (*S. persica*, *S. europea*, and *S. bigelovii*) under non-saline, moderate (300 mM NaCl), and high (500 mM NaCl) salinity conditions at three sampling times (1, 3, and 8 days after starting the treatments). Abbreviations: proline (PRO); glycine betaine (GB); malondialdehyde (MDA); hydrogen peroxide (H_2_O_2_); superoxide dismutase (SOD); catalase (CAT); peroxidase (POD); ascorbate peroxidase (APX).

				Mean Squares					
	df	PRO	GB	MDA	H_2_O_2_	SOD	CAT	POD	APX
Species	2	0.46 **	6304 **	0.65 **	0.06 *	25.94 **	1.88 **	338.41 **	1.04 **
Salinity	2	12.16 **	77,777 **	21.30 **	1.64 **	6828.96 **	114.59 **	14,595 **	18.60 **
Time	2	3.88 **	1527 **	2.70 **	0.11 **	65.91 **	3.31 **	366.75 **	2.99 **
Species × Salinity	4	0.19 **	935 **	0.10 **	0.04 *	65.55 **	1.36 **	22.73 *	0.13 **
Species × Time	4	0.47 **	524 **	0.40 **	0.009 ^ns^	6.81 *	0.28 ^ns^	23.84 *	0.03 ^ns^
Salinity × Time	4	1.27 **	1133 **	0.99 **	0.63 **	19.79 **	0.65 **	110.32 **	0.73 **
Species × Salinity × Time	8	0.21 **	361 *	0.18 **	0.02 *	4.40 ^ns^	0.37 **	15.49 *	0.02 *
Residuals	54	0.01	139	0.02	0.01	2.16	0.13	7.37	0.01
CV%		9.33	6.22	9.39	6.89	5.37	6.24	5.56	4.43

^ns^: Non-significant; *: Significant at *p* < 0.05; **: Significant at *p* < 0.01.

## Data Availability

Data are available within the article or Supplementary Material.

## Supplementary Materials

The following supporting information can be downloaded at: https://www.mdpi.com/article/10.3390/plants13070979/s1, Table S1: Eigenvectors and eigenvalues of the first two components of principal components analysis for the variables measured in three *Salicornia* species (*S. persica*, *S. europea*, and *S. bigelovii*) under non-saline, moderate (300 mM NaCl), and high (500 mM NaCl) salinity conditions during three sampling times (1, 3, and 8 days after applying salinity). Abbreviations: fresh weight (FW); water content (WC); chlorophyll a (Chla), chlorophyll b (Chlb), carotenoids (Car); hydrogen peroxide (H_2_O_2_); malondialdehyde (MDA); glycine betaine (GB); proline (PRO); peroxidase (POD); catalase (CAT); superoxide dismutase (SOD); ascorbate peroxidase (APX); sodium (Na^+^); potassium (K^+^); calcium (Ca^2+^); magnesium (Mg^2+^).
