# Supplementary material for: Temporal Changes in Biochemical Responses to Salt Stress in Three Salicornia Species
Source: Plants (Basel). 2024 Mar 29;13(7):979. doi: 10.3390/plants13070979 (PMC11013812; doi:10.3390/plants13070979)
Supplement: Supplementary file 1 [file plants-13-00979-s001.zip › plants-2918658-supplementary.pdf]

Supplementary Table S1. Eigenvectors and eigenvalues of the first two components of principal components analysis for the variables measured in three *Salicornia* species (*S. persica, S. europea*, and *S. bigelovii*) under non-saline, moderate (300 mM NaCl), and high (500 mM NaCl) salinity conditions during three sampling times (1, 3, and 8 days after applying salinity). Abbreviations: fresh weight (FW); water content (WC), chlorophyll a (Chla), chlorophyll b (Chlb), carotenoids (Car); hydrogen peroxide (H_2O_2); malondialdehyde (MDA); glycine betaine (GB); proline (PRO); peroxidase (POD); catalase (CAT); superoxide dismutase (SOD); ascorbate peroxidase (APX); sodium (Na^+); potassium (K^+); calcium (Ca^{2+}), and magnesium (Mg^{2+}).

Variable	Principal component	
	1	2
POD	0.973	-0.038
CAT	-0.935	-0.124
SOD	0.922	-0.223
APX	0.936	0.180
H_2O_2	0.605	0.355
GB	0.878	0.413
PRO	0.819	0.360
MDA	0.915	0.172
K^+	-0.911	0.101
Na^+	0.974	0.031
Ca^{2+}	0.701	-0.170
Mg^{2+}	-0.946	-0.235
FW	-0.011	0.928
WC	-0.105	0.931
Chla	-0.925	0.322
Chlb	-0.804	0.546
Car	-0.871	0.310
Eigenvalue	12.611	3.748
Proportion (%)	54.832	16.297
Cumulative (%)	54.832	71.130
